# Capturing the Unsaid: Nurses’ Experiences of Identifying Mental Ill-Health in Older Men in Primary Care—A Qualitative Study of Narratives

**DOI:** 10.3390/nursrep11010015

**Published:** 2021-03-04

**Authors:** Jenny Karlsson, Lena Marmstål Hammar, Birgitta Kerstis

**Affiliations:** 1 Centre for Psychiatry Research, Department of Clinical Neuroscience, Karolinska Institute, Stockholm Health Care Services, SE 113 64 Stockholm, Sweden; 2 School of Health, Care and Social Welfare, Malardalen University, SE-722 18 Vasteras, Sweden; lena.marmstal.hammar@mdh.se; 3 School of Education, Health and Social Studies, Dalarna University, SE-791 31 Falun, Sweden; 4 Department of Neurobiology, Care Sciences and Society, Karolinska Institute, SE-141 83 Huddinge, Sweden

**Keywords:** content analysis, experiences, men, mental ill-health, nurses, older people, primary healthcare, suicide

## Abstract

This study describes nurses’ experiences in identifying mental ill-health in older men in primary care. The aging population is growing in Sweden and life expectancy is increasing. Age is a risk factor for mental ill-health. Older men are over-represented in deaths from suicide. When older men seek primary care, it is often because of somatic symptoms and rarely for mental health issues. A questionnaire with five open questions was answered by 39 nurses from 10 primary care centres and subjected to inductive qualitative content analysis. The results revealed a main theme—capturing the unsaid—and two categories: (1) feeling secure in the role, with three subcategories (building trust, daring to ask and interpreting signs); and (2) the need for resources, with two subcategories (time and continuity, and finding support in collaboration). The results confirm that nurses in primary care play a key role in identifying mental ill-health in older men. There is a need for resources in the form of time, competence and collaboration with other professionals and patients’ relatives. This strategy will establish best practice and provide evidence-based care to facilitate improvements in older men’s mental health and prevent suicide.

## 1. Introduction

The World Health Organization (WHO) defines health as “a state of complete physical, mental and social well-being and not merely the absence of disease or infirmity” [1] (p. 7). Mental ill-health is described as a spectrum, from mild mental ill-health to severe mental illness [2]. The WHO states that mental ill-health will be the biggest global cause of illness for the world’s population by 2030 [3]. This is a growing challenge for society in general and especially for healthcare systems, including primary care.

According to the WHO, mental ill-health among older people is not sufficiently identified by either healthcare professionals or older people themselves [3]. Stigmas around mental ill-health can make people more unwilling to seek help for this disease compared with other kinds of diseases. This attitude makes it challenging for nurses in primary care to detect, support and treat mental ill-health in older persons. Primary care could be provided to many older people who experience mental ill-health more cost-effectively than specialist medical care [4].

A Swedish survey of a random sample of the Swedish population aged from 65 to 80 years showed that 25% of the cohort self-reported loneliness and 10% described depressive symptoms, and contrary to many other studies it revealed that depressive symptoms were more common in men than in women [5]. These findings highlight the importance of identifying loneliness and depressive symptoms to offer adequate support and treatment to improve well-being. Worldwide, the rate of depression among women is about twice as high as that among men [1,6]. However, far more men, especially older men, die from suicide [7]. In Sweden, older males have the highest suicide rates of all age groups [8]. Therefore, more research is needed in this group so that mental ill-health can be detected, and the required levels of support and treatment can be provided.

The difference between men and women regarding depression appears to relate to gendered expressions [9]. Men are also less likely than women to seek professional help or talk about mental health issues with family and friends [10]. Gender differences can also be seen in the prescription of antidepressants, where women are dominant. This observation is consistent with the fact that women are more often diagnosed with depression and may even be over diagnosed [10,11], whereas men risk being underdiagnosed. It is of note that these differences may be due, at least in part, to the screening tools used because they are designed for female expressions of depression, whereas men exhibit other symptoms [11]. Previous research suggests that symptoms in men might instead manifest through irritableness, anger, addiction, and flight behaviour [12]. Masculine norms can conflict with signs of depression, create feelings of shame that can inhibit help-seeking and reinforce maladaptive coping patterns [13].

During the transition to retirement, it can be difficult to both accept and adapt to new living conditions [14,15]. Older people in Finland and Sweden describe a life crisis that can cause deterioration or a poor quality of life (QOL) [16]. Older adults are vulnerable in society, which underlines the importance of a well-developed and inclusive welfare and social security system [17]. It is common that persons aged 65 years and older are not involved in digital society, which can lead to exclusion and affect everyday life [18].

Previous research has revealed that older people have limited trust when seeking help for mental ill-health and express a need for more knowledge from and better communication with healthcare providers [9]. Nurses in primary care are commonly the first in line to meet persons seeking treatment, and there is a need for nurses to increase their knowledge about mental ill-health so that they can identify, support, and offer treatment to such patients. An identified area of deficiency is a lack of education and/or experience in how to connect with patients with mental ill-health [19,20]. Nurses in primary care sometime lack communication skills and are insecure when caring for patients with mental ill-health [21,22]. In Sweden, there is no nationally developed or regulated conversational training during nursing education, so nurses may feel insecure about how to communicate with certain patients in a suitable way [23]. This is especially important for nurses in primary care. Nurses are uniquely situated to identify and provide care for persons with mental ill-health, and thus, should have the prerequisites necessary to identify mental ill-health, promote mental health and improve well-being and QOL [24]. The ability to identify mental ill-health and apply this knowledge in suicide prevention is of great importance. Nurse practitioners in primary care are ideally placed to recognize mental ill-health and use this knowledge successfully to prevent suicide [25]. As older men are overrepresented in suicide rates in Sweden, there is a need for greater knowledge about how to interact with this group to be able to identify, support and treat them. Therefore, the aim of the present study was to highlight primary healthcare nurses’ experiences identifying mental ill-health in older men in Sweden.

## 2. Materials and Methods

A qualitative descriptive design was used, where written narratives from nurses in primary care were subjected to qualitative content analysis.

### 2.1. Context

Primary care in Sweden supplies the population with healthcare for basic needs [26] and is responsible for the assessment and treatment of mild mental ill-health [27]. The participating nurses worked at one of 10 either urban or rural health centres located in a medium-sized region in the central part of Sweden.

### 2.2. Participants and Procedures

The deputy project manager in a county council in the central part of Sweden received oral and written information and approved participation in the study. Consequently, 11 nursing unit managers of public care centres were contacted, of whom, 10 agreed to participate. The unit managers provided the nurses’ email addresses, and subsequently, 134 nurses were contacted. Thirty-nine nurses (2 men and 37 women) participated (Table 1). The inclusion criteria were registered nurses, including undergraduates, and those with nursing degrees in primary care with experiences of meeting older men with mental ill-health.

### 2.3. Data Collection

A qualitative online survey was used as the data collection method in the Artologik software web tool [28]; the survey consisted of five open questions (Table 2). The nurses were instructed to share their experiences about identifying mental ill-health in older men in primary care settings by answering a few open-ended questions and with a clear information about the aim of the study, making the participants informative in their descriptions. The survey questions were originally in Swedish and later translated into English.

A total of 11 pages of text were collected and analysed.

### 2.4. Data Analysis

The written narratives were analysed following the steps for manifest qualitative content analysis as described by Graneheim and Lundman [29]. The analysis was performed by the first and the last authors (JK and BK), and in the first step, they separately read the narratives several times to get a grasp of the whole situation. Secondly, meaning units, such as words, sentences or sections related to the study aim, were identified in the text. Next, these were condensed, meaning that superfluous words were removed without changing the meaning of the text. The condensed text was then provided with a code that established the content of the text. Codes with similar content were then sorted into categories and subcategories. Categories have a higher level of abstraction than subcategories, and codes are more textual and concrete [29,30]. The authors that performed the analysis (JK and BK) then discussed their findings with the second authors to increase the trustworthiness of the analysis [31]. Any disagreement within the group was discussed until agreement was achieved. The content analysis was manifested, in the sense that it was mainly descriptive with minimal interpretation of the text except for the theme, which focused on the underlying meaning of the nurses’ experiences, in line with Graneheim and Lundman. During the whole analysis, discussions between the authors increased the level of credibility [31] so that the interpretations and abstractions revealed a red thread establishing the main theme [29], exposing the underlying meaning. Examples of the steps used in the analysis are provided in Table 3. Trustworthiness was increased by thorough data collection procedures and analytical steps, and credibility was achieved by using a semistructured instruction guide when developing the questions in the questionnaire. Trustworthiness was also increased by cooperation in the analysis, as described in the method section. Guidelines and rules were used to ensure that no misinterpretations arose. We strove to bridle our preconceptions during the work, which entails setting aside one’s own assumptions and preconceptions about a phenomenon while studying it [32].

### 2.5. Ethical Considerations

The nurses were informed by email about the study aims and procedures and of their right to withdraw from the study at any stage without explanation. They were also informed that they consented to participate in the study by answering the web-based questionnaire. Data were treated confidentially, with no identification of which nurse was related to each quote. This study follows Swedish law (2003:460) [33] and is in accordance with the Declaration of Helsinki [34]. The study, including the questioning tool, was approved by the local university ethical committee.

## 3. Results

The analysis resulted in an overall theme—capturing the unsaid—which was related to two categories: (1) feeling secure in the role, and (2) the need for resources (Table 4).

### 3.1. Capturing the Unsaid

The nurses’ experiences with identifying mental ill-health in older men highlighted several aspects that stemmed from feeling secure in the professional role, such as the importance of building trust, daring to ask questions, and interpreting signs. It also emerged that time, continuity and collaboration with other professions and relatives were important resources to identify mental ill-health. The nurses described that they needed a sensitive ear, as older men rarely explicitly stated that they experienced mental ill-health during nurse–patient communication. Rather, it was common for older men to present to primary healthcare for physical symptoms. The nurses were challenged to investigate by problematizing the symptoms the men described and at the same time being responsive to symptoms that were not clearly stated but could be the result of mental ill-health.

### 3.2. Feeling Secure in the Role

The data suggest that while identifying mental ill-health in older men was complex, experience and competence made it a bit less so. The findings revealed the importance of nurses feeling secure in the professional role, and this was linked to both formal and informal competencies that could be achieved through experience. Extensive clinical practice and competence were highlighted as essential for building trust and daring to ask questions, and the ability to interpret signs was described as crucial for identifying mental ill-health in older men.

#### 3.2.1. Building Trust

The nurses highlighted the ability to create a trusting relationship in their encounters with older men as crucial for them to open up and talk about their feelings and inner thoughts. A trusting relationship helped men open up and let the conversation deepen, which facilitated the identification of the underlying reasons for the contact.

*“It can happen in any patient encounter, if the contact is there and the patient feels confident to tell.” (D.10)*

The nurses described that they tried to create a safe environment, for example, by talking about everyday things, not just the specific reason for the visit, and they needed to listen actively with interest.

*“To try to create a safe climate where it is ok to tell. To listen to the unspoken and then curiously but respectfully continue to explore the mental health.” (D.33)*

The nurses also identified the value of being emotionally present in the meeting by listening actively to both verbal and nonverbal communication. Not only to capture what was said, but also to pay attention to what was not said and thus be responsive to the men’s need for support. Establishing a trusting relationship also made it easier for the nurses to dare to ask questions about mental ill-health, even when it felt difficult and uncomfortable.

#### 3.2.2. Daring to Ask Questions

The nurses described how older men rarely contacted primary care for pronounced mental ill-health issues. They identified that nurses needed to be responsive and dare to ask questions, while at the same time, being responsive to the older men’s way of communication when trying to detect mental ill-health.

*“It’s rare for them to talk about how they feel emotionally. But it can often be noticed that there is something that “chafes”. By asking and daring to stay in the conversation and listen behind what is said, there is quite often a loneliness and difficulty sleeping. A sadness that his wife has fallen ill or died. But my experience is that it’s often difficult to formulate for many older men, so it is like you have to help them along the way.” (D.34)*

It emerged that nurses’ experiences were that older men often had difficulty articulating their emotional health, which made it especially important to dare to ask questions about mental health even though it could feel uncomfortable as a sensitive topic for the nurses to bring up. Furthermore, it was recognised that when questions were asked, the nurses needed to stop and listen carefully to the answers and continue the conversation by asking supplementary questions, even if it felt awkward. “Fingertip feeling”, described as an intuition, helped the nurses determine how direct or gentle the questions should be. Equally important was how the nurses received the answers. Being exploratory in both posing the right questions and listening to the answers facilitated the ability to see behind the words.

*“To try to listen to the unspoken, to look at body language and to dare to ask”. (D.3)*

The nurses described earlier experiences with other older men as being of great value when meeting in caring situations. These experiences gave the nurses the confidence to discuss sensitive issues such as loneliness, alcohol and/or drug consumption and/or thoughts of not wanting to live.

#### 3.2.3. Interpreting Signs

It was common for contact with older men to take place over the phone, where an assessment was required in a short period of time. The nurses described that interpreting signs over the phone was more difficult compared with an actual meeting. The physical meeting facilitated attention to nuances in expressions, both verbal and nonverbal language that were obscured in phone conversations consisting of only spoken words. The nurses indicated that it was more difficult to have a deeper conversation over the phone than when meeting face-to-face, which contributed to the complexity of recognising and interpreting elusive signs.

*“I need to read between the lines.” (D.19)*

It also emerged that mental ill-health in older men was often identified when the older men presented for a physical affliction. The nurses emphasised that mental ill-health often emerged in a vague way, and that they needed to be responsive to both the spoken and unspoken words. This requirement placed high demands on their competence and experience in identifying mental ill-health.

*“Feel the need for competence to increase in all professions. That patients should not automatically meet a counsellor or psychologist when someone shows signs of mental ill-health. I think a nurse could do it quite well.” (D.20)*

Several of the nurses pointed out the need for increased knowledge in this regard.

*“Heard, noticeable, feels. The longer you work, the easier it gets.” (D.28)*

Nurses needed to pay attention to older men’s changing search patterns; for example, that they were contacting the healthcare setting more often and for various diffuse symptoms, as it “could be” an indication of mental ill-health. Furthermore, the nurses described that older men sometimes found it difficult to identify and verbalize that their problem was mental ill-health. The nurses described that a suspended driving licence because of an examination of memory problems could be a major blow that could affect an older man’s mental health. The nurses emphasised that it was important to be sensitive to the older men’s reactions in connection with such changes.

### 3.3. The Need for Resources

Time, continuity and collaboration with other professions and relatives emerged as important prerequisites that facilitated the identification of mental ill-health in older men. To be able to identify underlying mental ill-health, the nurses needed to be curious in a process sometimes described as detective work, where time and continuity were emphasised as prerequisites and presupposed a stable collaboration with the older men’s relatives, as well as with internal and external professionals.

#### 3.3.1. Time and Continuity

Time and continuity were described as important when identifying mental ill-health in older men. Above all, insufficient time was described as an obstacle that made it difficult to identify mental ill-health.

*That they have difficulty talking but that they may need it just as much. That healthcare does not have time to wait. That we’re missing it because of a lack of time. (D.1)*

The nurses emphasised that a lack of time could lead to patients avoiding questions about mental ill-health and instead just focusing on the specific physical complaint. Furthermore, they described that older men sometimes had difficulty talking about mental ill-health, and that time was a prerequisite to create the opportunity for men to dare to start talking about possible underlying mental ill-health. The nurses emphasised the importance of continuity, namely that meeting the same person several times created security in the relationship between the older men and the nurses, which facilitated talking about underlying mental ill-health.

*“As a nurse in primary care, there is rarely so much time spent per visit and we are not expected to take that role, which I might find sad. I would have liked to have had more conversations about life in general.” (D.33)*

Time and continuity in the contact with the older men helped the nurses identify life changes in the older men’s lives that could cause mental ill-health. The identification of underlying mental ill-health was described as detective work where time and continuity were highlighted as prerequisites.

#### 3.3.2. Collaboration with Professions and Relatives

Collaboration with the older men’s relatives as well as with internal and external professionals such as psychiatrists was described as important throughout the care process.

*“Seldom do the man describe how he feel emotionally, so I get information from the wife or the children about their interpretations of the man’s mental health.” (D.12)*

This could include contact with professionals with greater experience of working with mental ill-health.

*Psychotherapists have developed a cheat sheet to use in the work with patients with mental health problems. This has been extremely helpful. (D.1).*

The nurses indicated that the most common arrangement after the identification of mental ill-health was that they referred the older men to another professional, such as a physician, psychologist, or counsellor. If signs of serious mental illness and/or suicidal thoughts were detected, the patient was referred to a psychiatrist. A contributing part of the ongoing care of older men in primary care was described as consisting of assessments, information, collaboration and referral to other professionals and levels of care. Relatives were important for facilitating the identification of mental ill-health. It was common that relatives contacted primary care nurses because they were worried about their husband or relative’s health and/or behaviour. Behavioural changes could include increased irritation, lack of appetite or a more general perception that something was wrong. The next of kin was an important resource describing the changes they had noticed. They could also be helpful and supportive in motivating the older men to seek help.

*If I know through relatives that they (the older man) do not feel well, I usually start by asking how they feel and if they only talk about physical symptoms. I can approach the topic by telling them that it is common to feel depressed for various reasons, … but for the most part, they do not describe how they feel, so what I must go on is the wife’s or the children’s interpretations of the man’s mental health situation. (D.12)*

## 4. Discussion

The aim of this study was to highlight nurses’ experiences identifying mental ill-health in older men in primary care. The findings stress the complexity of identifying mental ill-health, which is in line with previous research [5] suggesting that men’s symptoms can differ from standard classifications, which makes identification difficult [12]. The nurses described that men often presented physical symptoms even if the issue was psychological. They emphasised that mental ill-health often emerged in a diffuse way and was not communicated clearly, which can be seen in the light of the overall theme: capturing the unsaid. The nurses needed to be responsive and attentive to nonverbal communication and to have knowledge of the varying ways of expression of mental ill-health. Furthermore, the nurses described the importance of creating a trusting and open climate to make the men more comfortable to talk about mental ill-health issues. Thus, a trusting relationship was a prerequisite, as delineated in the theory of person-centred care. All care should be person-centred and this may be especially important when working with vulnerable persons [35], as it might prevent suffering and even suicide.

Our results revealed that enhancing a trusting relationship, being able to identify signs of mental ill-health, time and continuity were essential. Being able to have sufficient time and continuity in meetings was also described as a facilitator to deepen conversations and identify changes in a man’s sense and behaviour that may indicate mental ill-health. As the nurse–patient relationship is of utmost importance in person-centred care, continuity is crucial. This recommendation accords with previous research that highlights the problem with time pressure and low continuity in primary care as barriers when it comes to recognizing mental ill-health and preventing suicide [36].

According to our results, the nurses highlighted the importance of asking about mental health issues even if it was a sensitive topic for the nurses to ask about, especially if the man had been seeking help for a physical symptom. Having the courage to ask questions was linked to work experience and a sense of being confident in the professional role. This is in accordance with descriptions of the importance of nurses feeling safe when encountering patients with mental ill-health [37]. They also stressed the importance of general competence and training regarding mental health issues. This is consistent with a previous study [12] that described a need for professional competence and training to detect depression in older men, including the ability to identify signs of mental distress in body language and voice modes.

Overall, our findings emphasize that nurses in primary care are important for identifying mental ill-health among older men but are dependent on organisational conditions such as time and continuity. Age and normative gender patterns probably lead to underdiagnosing men’s mental ill-health, which confirms the findings of previous research [5,9,38,39]. Therefore, it is relevant to investigate further the impact that masculine norms may have on men’s mental health and help-seeking patterns. Based on the complexity of this focus, we suggest that nurses need to gain more knowledge about aging, gender and norms and also need training in communication, meeting and identifying people with mental ill-health as indicated in our results. This is in line with previous research [37] emphasizing that knowledge and training in mental health makes nurses more confident and positive in their role when meeting and treating patients with mental-ill-health. In addition, it is important to raise awareness of mental health problems in society in general to not only minimize any associated taboos, but also target older persons, with a specific focus on older men. Older men’s mortality may be related to poor mental health, including suicide [40], and be problematic to diagnose because they are not used to talking about their mental health problems, as described in our study.

The need for a greater knowledge of mental ill-health has been described in previous research [21,22], where it appears that nurses in primary care feel insecure regarding mental health problems and have little educational training in this regard. This is consistent with our findings, which revealed that several nurses felt insecure in identifying mental ill-health in older men and described a need for more knowledge and training regarding mental health issues. The Swedish National Board of Health and Welfare recognizes that healthcare professionals often need more knowledge when it comes to detecting, treating, responding to, and preventing mental health problems in older people [4]. A lack of knowledge can thus be a reason why mental ill-health is difficult to identify in primary care, especially since the symptoms of mental ill-health in older people can be diffuse. The results of our study increase this knowledge by shedding light on this matter, namely that older men may search healthcare for other reasons. Nurses usually have adequate knowledge to observe and assess a patient’s physical status [23], but they often perceive it as more difficult to detect mental ill-health. This accords with our findings, where several nurses found it more convenient to discuss physical issues because it was easier to focus on specific symptoms and more difficult to identify, ask and talk about mental health issues; this could be related to the lack of systematic conversational training during nursing education in Sweden [23]. Having a psychiatric team with mental health nurse specialists could increase competencies regarding mental ill-health in primary care settings, as suggested by Leavey et al. [36]. The Swedish government report *Good and Close Care* further states that primary care’s basic assignments are both physical and mental health. The report emphases the importance of specialist nurses in primary care for identifying mental ill- health and meeting patients’ needs [26]. Based on our results we also stress the importance of collaboration with other professions and patients’ relatives to identify mental ill-health in older men. This is in accordance with a Swedish study that states that identifying adult patients’ mental ill-health is time- and energy-consuming and requires teamwork [41].

### Strengths and Limitations

There are several strengths and weaknesses in this study. A strength of the data collection procedures is the use of the written narratives, where there is no need to participate physically, facilitating more participation. However, in contrast to interviews, written text precludes further explorative questions, which probably would have made the data more in depth, as the interviewer then can encourage the participants to develop their answers. Credibility and robustness were also enhanced by the fact that the nurses independently expressed similar outlines in their descriptions. Dependability was increased as the narratives were independently read and analysed by the researchers with different backgrounds, such as a mental health nurse, a dementia nurse, and a primary care nurse, and the manuscript was discussed during a seminar where input of other researchers and nurse practitioners was considered.

A total of 39 nurses responded to the survey, which was a low response rate (29%). However, according to Graneheim et al. [30], the worth of research data is not determined by the number of participants, but rather, by its quality. The data was rich enough to be analysed. Another limitation was that most of the participants in our study were women (95%). However, the gender distribution in our study is in line with the gender distribution among nurses in primary care in Sweden, justifying our sample. The Swedish context should be considered regarding transferability. However, our results confirm other findings in this area and could be expected to strengthen the knowledge of nurses and other staff in primary care, as older men’s mental health is an urgent and important topic.

## 5. Conclusions

This study contributes to the awareness of nurses’ experiences identifying mental ill-health in older men in primary healthcare and highlights the complexity in this field. To identify mental ill-health nurses in primary care need competence, experience, to feel secure in the role, time, continuity, and the ability to collaborate. We emphasize that primary care nurses have a key position in identifying mental ill-health among the entire population, especially older men, who often visit primary care for a physical reason when there may be an underlying mental ill-health issue. Therefore, we recommend that primary care nurses be allocated sufficient resources in the form of more time during patient visits, the ability to maintain continuity and training to identify mental health issues in the entire population, including older men. This would ensure that older men’s mental ill-health is sufficiently identified by nurses in primary healthcare and that suitable treatment would ensue with a concomitant reduction in suicide. Primary healthcare is the foundation of the Swedish healthcare system and is therefore ideal for identifying mental ill-health and initiating the right interventions. Compared with specialist medical care, it is also a cost-effective care option for increasing the well-being and QOL of individuals and their relatives.

## Figures and Tables

**Table 1:** The nurses’ demographic data.

Nurses *N* = 39	
*Component*	
**Professional**	
District nurse *n* (%)	29 (74)
Nurse *n* (%)	9 (23)
Diabetic nurse *n* (%)	1 (3)
**Sex**	
Female *n* (%)	37 (95)
Male *n* (%)	2 (5)
**Age** mean (SD)	50 (10)
**Work experiences** mean (SD)	21 (12)

**Table 2:** Survey questions about the nurses’ experiences identifying mental ill-health in older men.

Survey Questions
What professional title do you have in your employment? What experiences do you have as a nurse in primary care to discuss mental health issues with older men? (aged 65 years and older) Describe how you as a nurse identify mental ill-health in older men. Describe how you as a nurse continue to ask when you have identified mental ill-health in older men. Do you have other experiences regarding older men and mental ill-health issues that have not been given space in the previous questions?

**Table 3:** Examples of steps in the analysis.

	Condensed Sentence Unit	Code	Subcategories	Category	Theme
During health calls made when measuring blood pressure, they can start to talk about it, even if they are looking for sleep problems	During health calls when measuring blood pressure, they can talk about it	Emerges at meetings about physical symptoms	Interpreting signs	**To feel secure in the role**	**Capturing the unsaid**
Can really happen in any patient meeting if the contact is there and the patient feels confident to tell	Can happen in any patient meeting if contact and trust exist	If trust exists	Building trust		
Time is needed, and it is easier if you have met on several occasions	Time needed, easier if met several times	Time needed and multiple meetings	Time and continuity	**To need resources**	
Most often, relatives are the ones who make contact and are worried	Relatives get intouch for concern	Relatives get in touch	Finding support in collaboration		

**Table 4:** Overview of the subcategories, categories, and main theme.

Subcategories	Categories	Main Theme
Building trust Daring to ask Interpreting signs	To feel secure in the role	Capturing the unsaid
Time and continuity Collaboration with other professions and relatives	To need resources	

## Data Availability

The data presented in this study are available in article.

## References

[1] World Health Organization (2017). Depression and Other Common Mental Disorders Global Health Estimates.

[2] The Public Health Agency of Sweden Our Mission—To Strengthen and Develop Public Health. https://www.folkhalsomyndigheten.se/livsvillkor-levnadsvanor/psykisk-halsa-och-suicidprevention/vad-ar-psykisk.

[3] World Health Organization (2017). Mental Health of Older Adults.

[4] The National Board Se Tecken och Ge Rätt Stöd i Tid (See Signs and Give Right Support in Time). https://www.socialstyrelsen.se/globalassets/sharepoint-dokument/artikelkatalog/ovrigt/vagledning-primarvard.

[5] Djukanović I., Sorjonen K., Peterson U.J. (2015). Association between depressive symptoms and age, sex, loneliness, and treatment among older people in Sweden. Aging Ment. Health.

[6] Luppa M., Sikorski C., Luck T., Ehreke L., Konnopka A., Wiese B., Weyerer S., König H.-H., Riedel-Heller S.G. (2012). Age-and gender-specific prevalence of depression in latest-life—Systematic review and meta-analysis. J. Affect Disord..

[7] Koo Y.W., Kõlves K., De Leo D. (2017). Suicide in older adults: Differences between the young-old, middle-old, and oldest old. Int. Psychogeriatr..

[8] Public Health Agency of Sweden Suicide and Suicide Prevention in Sweden. https://www.folkhalsomyndigheten.se/suicidprevention/statistik-om-suicid/#:~:text=H%C3%B6gst%20suicidtal%20fann.

[9] Rydberg Sterner T., Gudmundsson P., Falk H., Seidu N., Ahlner F., Wetterberg H., Rydén L., Sigström R., Östling S., Zettergren A. (2020). Depression in relation to sex and gender expression among Swedish septuagenarians—Results from the H70 study. PLoS ONE.

[10] Affleck W., Carmichael V., Whitley R. (2018). Men’s mental health: Social determinants and implications for Services. Can. J. Psychiatry.

[11] Thunander Sundbom L., Hedborg K. (2019). Association between prescribed antidepressants and other prescribed drugs differ by gender: A nationwide register-based study in Sweden. Nord. J. Psychiatry..

[12] Oliffe J.L., Rossnagel E., Seidler Z.E., Kealy D., Ogrodniczuk J.S., Rice S.M. (2019). Men’s Depression and Suicide. Curr. Psychiatry Rep..

[13] Seidler Z.E., Dawes A.J., Rice S.M., Oliffe J.L., Dhillon H.M. (2016). The role of masculinity in men’s help-seeking for depression: A systematic review. Clin. Psychol. Rev..

[14] World Health Organization (2014). Preventing Suicide. A Global Imperative.

[15] Suvanto S.R. (2012). Mellan Äldreomsorg och Psykiatri: Om Värd och Bemötande av Äldre med Psykisk Ohälsa, (Between Care for the Elderly and Psychiatry: About the Host and Treatment of the Elderly with Mental Illness.

[16] Glasberg A.L., Pellfolk T., Fagerström L. (2014). Zest for life among 65-and 75-year-olds in Northern Finland and Sweden—A cross-sectional study. Scand. J. Caring Sci..

[17] Dahlberg L., McKee K.J. (2014). Correlates of social and emotional loneliness in older people: Evidence from an English community study. Aging Ment. Health.

[18] Reneland-Forsman L. (2018). ‘Borrowed access’—The struggle of older persons for digital participation. Int. J. Lifelong Educ..

[19] Björkman T., Angelman T., Jönsson M. (2008). Attitudes towards people with mental illness: A cross-sectional study among nursing staff in psychiatric and somatic care. Scand. J. Caring Sci..

[20] Waidman M.A.P., Marcon S.S., Pandini A., Bessa J.B., Paiano M. (2012). Nursing care for people with mental disorders, and their families, in primary care. Acta Paul. Enferm..

[21] Burroughs H., Lovell K., Morley M., Baldwin R., Burns A., Chew-Graham C. (2006). ‘Justifiable depression’: How primary care professionals and patients view late-life depression? A qualitative study. Fam. Pr..

[22] Haddad M., Plummer S., Taverner A., Gray R., Lee S., Payne F., Knight D.J. (2005). District nurses’ involvement and attitudes to mental health problems: A three-area cross-sectional study. Clin. Nurs..

[23] Bullington J., Söderlund M., Sparén E.B., Kneck Å., Omérov P., Cronqvist A. (2019). Communication skills in nursing: A phenomenologically based communication training approach. Nurse Educ. Pr..

[24] Dardas L.A., Bailey D.E., Simmons L.A. (2016). Adolescent depression in the Arab region: A systematic literature review. Issues Ment. Health Nurs..

[25] Raue P.J., Ghesquiere A.R., Bruce M.L. (2014). Suicide risk in primary care: Identification and management in older adults. Curr. Psychiatry Rep..

[26] Goverment Office of Sweden (2021). God och Nära Vård—Rätt Stöd till Psykisk Hälsa (Good and Close Care–Right Support for Mental Health) SOU 2021:6. https://www.regeringen.se/rattsliga-dokument/statens-offentliga-utredningar/2021/01/sou-20216/.

[27] Goverment Office of Sweden (2017). National Board of Health and Welfare. https://www.government.se/government-agencies/national-board-of-health-and-welfare--socialstyrelsen/.

[28] The Artologik Software Smart, Web-Based Programs for Cloud Computing Services. https://www.artologik.com/en/Start.aspx.

[29] Graneheim U.H., Lundman B. (2004). Qualitative content analysis in nursing research: Concepts, procedures and measures to achieve trustworthiness. Nurse Educ. Today.

[30] Graneheim U.H., Lindgren B.-M., Lundman B. (2017). Methodological challenges in qualitative content analysis: A discussion paper. Nurse Educ. Today.

[31] Creswell J. (2017). Qualitative Inquiry and Research Design—Choosing among Five Traditions.

[32] Dahlberg K., Dahlberg H., Nyström M. (2008). Reflective Lifeworld Research.

[33] Swedish Law. 2003:460. https://www.riksdagen.se/sv/dokument-lagar/dokument/svensk-forfattningssamling/lag-2003460-om-etik.

[34] World Medical Association (2013). World Medical Association Declaration of Helsinki Ethical Principles for Medical Research Involving Human Subjects.

[35] McCormack B., McCance T., Dewing J. (2020). The Person in Person-Centred Practice.

[36] Leavey G., Mallon S., Rondon-Sulbaran J., Galway K., Rosato M., Hughes L. (2017). The failure of suicide prevention in primary care: Family and GP perspectives—A qualitative study. BMC Psychiatry.

[37] Payne F., Harvey K., Jessopp L., Plummer S., Tylee A., Gournay K. (2002). Knowledge, confidence and attitudes towards mental health of nurses working in NHS Direct and the effects of training. J. Adv. Nurs..

[38] Danielsson U., Bengs C., Lehti A., Hammarström A., Johansson E.E. (2009). Struck by lightning or slowly suffocating–gendered trajectories into depression. BMC Fam. Pr..

[39] River J. (2018). Diverse and dynamic interactions: A model of suicidal men’s help seeking as it relates to health services. Am. J. Men’s Health.

[40] Kiely K.M., Brady B., Byles J. (2019). Gender, mental health and ageing. Maturitas.

[41] Janlöv A.C., Johansson L., Clausson E.K. (2018). Mental ill-health among adult patients at healthcare centers in Sweden: District nurses experiences. Scand. J. Caring Sci..

